# GSDS 2.0: an upgraded gene feature visualization server

**DOI:** 10.1093/bioinformatics/btu817

**Published:** 2014-12-10

**Authors:** Bo Hu, Jinpu Jin, An-Yuan Guo, He Zhang, Jingchu Luo, Ge Gao

**Affiliations:** ^1^State Key Laboratory of Protein and Plant Gene Research, College of Life Sciences, Center for Bioinformatics, Peking University, Beijing 100871, People’s Republic of China, ^2^College of Life Science, Beijing Normal University, Beijing 100875, People’s Republic of China and ^3^Department of Systems Biology, College of Life Science and Technology, Huazhong University of Science and Technology, Wuhan 430074, People’s Republic of China

## Abstract

**Summary**: Visualizing genes’ structure and annotated features helps biologists to investigate their function and evolution intuitively. The Gene Structure Display Server (GSDS) has been widely used by more than 60 000 users since its first publication in 2007. Here, we reported the upgraded GSDS 2.0 with a newly designed interface, supports for more types of annotation features and formats, as well as an integrated visual editor for editing the generated figure. Moreover, a user-specified phylogenetic tree can be added to facilitate further evolutionary analysis. The full source code is also available for downloading.

**Availability and implementation**: Web server and source code are freely available at http://gsds.cbi.pku.edu.cn.

**Contact**: gaog@mail.cbi.pku.edu.cn or gsds@mail.cbi.pku.edu.cn

**Supplementary information**: Supplementary data are available at *Bioinformatics* online.

## 1 Introduction

The visualization of gene features such as composition and position of exons and introns for genes offers visual presentation for biologists to integrate annotation, and also helps them to produce high-quality figures for publication. Thus, several web servers/software including FancyGene (Rambaldi and Ciccarelli, 2009), GECA (Fawal et al., 2012), FeatureStack (Frech et al., 2012), GSDraw (Wang et al., 2013), GPViz (Snajder et al., 2013) and GenePainter (Hammesfahr et al., 2013) have been developed recently. Designing to generate high-quality figures suitable for publication, we developed an online Gene Structure Display Server (GSDS) (Guo et al., 2007), which supported three input formats including sequences, accession number of GenBank (Benson et al., 2013) and exon positions. With more than 1 million hits annually, GSDS has been widely used by world-wide scientists in the functional (Ye et al., 2009; Wang et al., 2010) and evolutionary study (Hu et al., 2010; Yin et al., 2009; Yu et al., 2009) of gene families.

According to the feedbacks from GSDS users, we developed the upgraded GSDS 2.0. Compared with the previous version, GSDS 2.0 supports two more widely used annotation formats, providing more comprehensive support for annotation files. To aid biologists generating suitable figures for publication, GSDS 2.0 offers a powerful interactive interface. Users can customize the size, shape and color of annotation features after their initial render and even fine-tune each element through an integrated visual editor. To facilitate evolutionary analysis, a user-specified phylogenetic tree can be added to the figure. Finally, the generated figure can be exported as either vector graphic (in SVG and PDF format), or raster graphic (in PNG format).

## 2 Usage and implementation

**Fig. 1.**
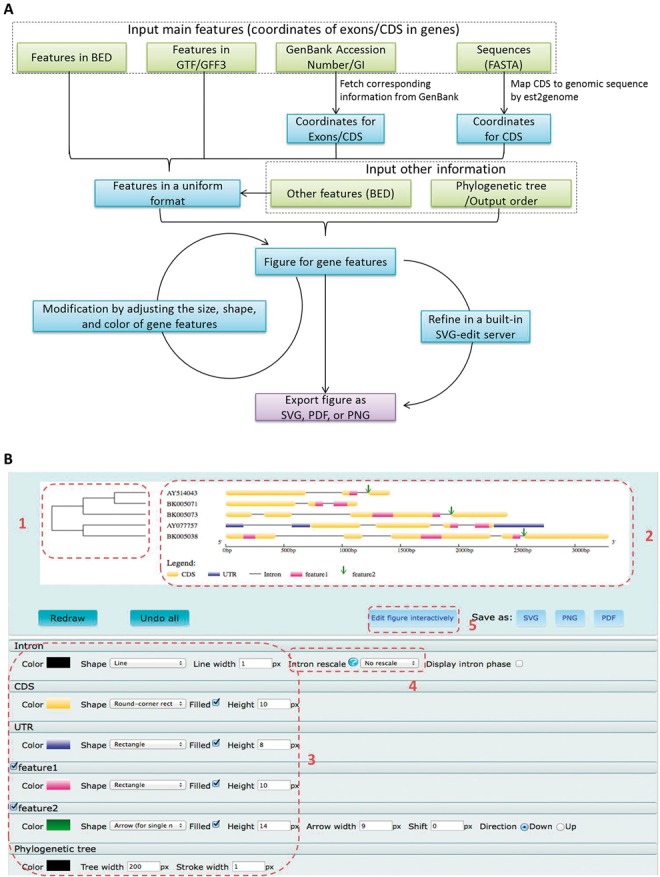
The work flow of GSDS 2.0 (**A**) and the generated figure (**B**). A user-specified phylogenetic tree (B1) could be added to the main canvas (B2). Meanwhile, the users can adjust the size, shape and color of all features (B3), rescale introns (B4), as well as edit generated figure interactively (B5)

Based on feedbacks from users of GSDS 1.0, we upgraded GSDS to version 2.0 with a newly designed interface and several novel functions (Fig. 1):

1. More types of annotation features and formats are supported. In addition to GenBank ID and FASTA sequences, GSDS 2.0 also supports commonly used formats including BED, GTF and GFF3. Moreover, extra features such as conserved elements and binding sites can also be uploaded and displayed along with the main features (‘Region 2’ in Fig. 1B).
2. The generated figures can be further modified by users. With the purpose to generate suitable, high-quality figures for publication, GSDS 2.0 provides types of functionality on their further modification (Fig. 1B). By setting the parameters on the result page (‘Region 3’ in Fig. 1B), users can turn on/off the display of specified features and adjust their size, shape and color. Intron lengths can also be rescaled to display large introns properly (‘Region 4’ in Fig. 1B). Moreover, the generated figure can be sent to a built-in online SVG editor for further modification interactively after clicking the ‘Edit figure interactively’ button (‘Region 5’ in Fig. 1B).
3. According to the published works using original GSDS 1.0, a large proportion of analyzes have been targeted on the evolutionary study of gene structures. Thus, we implemented a dedicated panel for users to specify a phylogenetic tree for display side-by-side with the main canvas (‘Region 1’ in Fig. 1B).

Compared with similar tools, GSDS 2.0 shows superiors in both the supports for input/output formats, usability and availability (Supplementary Table S1).

## 3 Further directions

We have updated our GSDS to version 2.0, which supports more types of features and feature describing formats, as well as further modification on the generated figures. By analyzing the usage of GSDS and feedbacks from users, we will continue our efforts to update GSDS to better serve this community.

## Supplementary Material

Supplementary Data
