# Supplementary material for: GSDS 2.0: an upgraded gene feature visualization server
Source: Bioinformatics. 2014 Dec 10;31(8):1296–7. doi: 10.1093/bioinformatics/btu817 (PMC4393523; doi:10.1093/bioinformatics/btu817)
Supplement: Supplementary Data [file supp_31_8_1296__index.html]

GSDS 2.0: an upgraded gene feature visualization server — GSDS 2.0: an upgraded gene feature visualization server — GSDS 2.0: an upgraded gene feature visualization server — Supplementary Data 

# GSDS 2.0: an upgraded gene feature visualization server

## Supplementary Data

files

**Files in this Data Supplement:**

- Supplementary Data - pdf file
