# Supplementary material for: GSDS 2.0: an upgraded gene feature visualization server
Source: Bioinformatics. 2014 Dec 10;31(8):1296–7. doi: 10.1093/bioinformatics/btu817 (PMC4393523; doi:10.1093/bioinformatics/btu817)
Supplement: Supplementary Data [file supp_btu817_Supplementary_Table_S1.pdf]

Supplementary Table S1. Comparison of features supported by GSDS 2.0 and other available tools. The feature supported by the tool is marked with “√”, otherwise marked with “-”.

Tool	Input format					Other feature			Further modification on generated figure		Output format		Availability	
	BED	GenBank ID	GFF3	GTF	Sequence	Other domain feature	Sequence alignment	Phylogenetic tree	Web-based	Built-in interactive editor	Vector graphic	Raster graphic	Online server	Open source
GSDS 2.0	√	√	√	√	√	√	-	√	√	√	√	√	√	√
FancyGene	-	-	√	√	-	√	-	-	√	-	√	√	√	-
FeatureStack	-	-	√	-	-	√	-	-	-	-	√	√	-	√
GECA	-	-	-	-	√	-	√	-	-	-	-	√	√	√
GenePainter	-	-	-	-	√	√	√	-	-	-	√	-	-	√
GSDraw	-	-	-	-	√	√	-	√	√	-	-	√	√	-
GPViz	√	-	-	√	-	√	-	-	-	√	√	√	-	√
